# Multicenter Analytical Validation of Aβ40 Immunoassays

**DOI:** 10.3389/fneur.2017.00310

**Published:** 2017-07-03

**Authors:** Linda J. C. van Waalwijk van Doorn, Luka Kulic, Marleen J. A. Koel-Simmelink, H. Bea Kuiperij, Alexandra A. M. Versleijen, Hanne Struyfs, Harry A. M. Twaalfhoven, Anthony Fourier, Sebastiaan Engelborghs, Armand Perret-Liaudet, Sylvain Lehmann, Marcel M. Verbeek, Eugeen J. M. Vanmechelen, Charlotte E. Teunissen

**Affiliations:** ^1^Department of Neurology, Radboud University Medical Center, Radboud Alzheimer Centre, Donders Institute for Brain, Cognition and Behaviour, Nijmegen, Netherlands; ^2^Department of Laboratory Medicine, Radboud University Medical Center, Radboud Alzheimer Centre, Donders Institute for Brain, Cognition and Behaviour, Nijmegen, Netherlands; ^3^Institute for Regenerative Medicine (IREM), University of Zurich, Schlieren, Switzerland; ^4^Neurochemistry Laboratory and Biobank, Department of Clinical Chemistry, VU University Medical Center, Neurocampus, Amsterdam, Netherlands; ^5^Reference Center for Biological Markers of Dementia (BIODEM), Institute Born-Bunge, University of Antwerp, Antwerp, Belgium; ^6^Neurobiology Laboratory, Centre for Memory Resources and Research (CMRR), Groupement Hospitalier Est (GHE), Hôpitaux de Lyon, Université Lyon 1, CNRS UMR5292, INSERM U1028, Lyon, France; ^7^Memory Clinic and Department of Neurology, Hospital Network Antwerp (ZNA) Middelheim and Hoge Beuken, Antwerp, Belgium; ^8^CHU de Montpellier and Université de Montpellier, IRMB, Laboratoire de Biochimie Protéomique Clinique, Montpellier, France; ^9^R&D, ADx NeuroSciences, Ghent, Belgium

**Keywords:** method validation, Alzheimer’s disease, cerebrospinal fluid, amyloid, Immunoassays

## Abstract

**Background:**

Before implementation in clinical practice, biomarker assays need to be thoroughly analytically validated. There is currently a strong interest in implementation of the ratio of amyloid-β peptide 1-42 and 1-40 (Aβ42/Aβ40) in clinical routine. Therefore, in this study, we compared the analytical performance of six assays detecting Aβ40 in cerebrospinal fluid (CSF) in six laboratories according to a recently standard operating procedure (SOP) developed for implementation of ELISA assays for clinical routine.

**Methods:**

Aβ40 assays of six vendors were validated in up to three centers per assay according to recently proposed international consensus validation protocols. The performance parameters included sensitivity, precision, dilutional linearity, recovery, and parallelism. Inter-laboratory variation was determined using a set of 20 CSF samples. In addition, test results were used to critically evaluate the SOPs that were used to validate the assays.

**Results:**

Most performance parameters of the different Aβ40 assays were similar between labs and within the predefined acceptance criteria. The only exceptions were the out-of-range results of recovery for the majority of experiments and of parallelism by three laboratories. Additionally, experiments to define the dilutional linearity and hook-effect were not executed correctly in part of the centers. The inter-laboratory variation showed acceptable low levels for all assays. Absolute concentrations measured by the assays varied by a factor up to 4.7 for the extremes.

**Conclusion:**

All validated Aβ40 assays appeared to be of good technical quality and performed generally well according to predefined criteria. A novel version of the validation SOP is developed based on these findings, to further facilitate implementation of novel immunoassays in clinical practice.

## Introduction

Cerebrospinal fluid (CSF) biomarkers have proven to be helpful in early diagnosis of Alzheimer’s disease (AD). They reflect preclinical early events in AD by as many as 10–15 years before clinical symptoms occur (1). Therefore, CSF biomarkers have been incorporated in the diagnostic criteria of AD (2–4).

The CSF biomarkers most prominently used in Alzheimer’s diagnostics are amyloid-β peptide 1-42 (Aβ42), total tau protein (t-tau), and tau phosphorylated at threonine 181 (p-tau), because they reflect the pathological hallmarks of AD (5, 6). A decrease in CSF Aβ42 levels probably reflects the extent of amyloid-β accumulation in the formation of plaques in the brain, while an increase in CSF t-tau and p-tau levels likely reflects neuronal degeneration and intracellular tangle formation. The ratio of Aβ42/Aβ40 is helpful to correct levels of Aβ42 for the total amyloid production (7) and provide better assessment of the presence of amyloid pathology in case of for instance cerebral amyloid angiopathy (8). For example, recent studies demonstrated that the CSF Aβ42/Aβ40 ratio significantly improved the diagnostic performance compared to CSF Aβ42 alone in distinguishing controls or non-AD patients from mild cognitive impairment or AD patients (9–13). The concordance with amyloid positron emission tomography increased when the CSF Aβ42/Aβ40 ratio was used as compared to CSF Aβ42 alone (14). Therefore, there is currently a strong interest to implement the Aβ42/Aβ40 ratio into clinical practice. However, before implementation, it is important to assess the quality of the Aβ40 assays and validation is an essential step in implementation of novel assays in clinical routine. In this study, we evaluated the performance parameters of six Aβ40 assays currently commercially available and one in-house assay according to an international consensus protocol following the ISO 15189 guidelines (15, 16). The results will provide insight into the real-life use of this standard operating procedure (SOP) for implementation of novel immunoassays in clinical routine. Moreover, we determined the inter-laboratory variation and compared the quality and outcomes of the ELISA assays.

## Materials and Methods

### CSF Samples

The inter-laboratory variation was tested by using 20 CSF samples centrally distributed in aliquots by one center. The samples were shipped on dry ice and upon receipt stored at −80°C in all laboratories. These samples were patient samples from an outpatient clinic, mostly patients who came for dementia diagnostic screening. Participants or their legal representatives gave informed consent. The study conforms with The Code of Ethics of the World Medical Association (Declaration of Helsinki) (17). Furthermore, for all the other parameters, samples were used that were available in each of the laboratories participating in this study. The samples were diluted according to protocols provided by the manufacturers (Table 1).

**Table 1:** Overview of Aβ40 assays tested in different laboratories.

Vendor	Kit	Cat (#)	Read-out	Standards (#)	Controls (#)	Sample dilution (factor)	Standard range (pg/mL)	Lab #1	Lab #2	Lab #3	Lab #4	Lab #5	Lab #6
MSD	Aβ peptide panel I	K15199E	Electrochemiluminescence	8		2×	4–15,000	x	x		x		
	(4G8)												
IBL	Amyloid-β (1-40)	RE59651	Optical density	6	2	20×	0–1,900	x		x	x		
	CSF ELISA												
Invitrogen	ELISA kit	LNB0001	Optical density	8		4×	0–500		x			x	x
	Human Aβ40												
Novex	Human Aβ40	LHB3481	Luminescence	8		2×	0–5,000					x	x
	Singleplex bead kit												
Fujirebio	INNOTEST	81585	Optical density	6	2	100×	5–1,000	x	x			x	
	β-amyloid (1-40)												
Euroimmun	β-amyloid	EQ 6511-9601-L	Optical density	6	2	21×	0–800			x	x		x
	(1-40) ELISA												
VUmc	In-house Aβ40	(18)	Optical density	8	2	25×	0–25,000	x					

*Aβ, amyloid-β; Cat (#), catalogus number; CSF, cerebrospinal fluid; ELISA, enzyme-linked immunosorbent assay; IBL, IBL-international; MSD, Meso Scale Discovery; VUmc, VU university medical center*.

### Participants and Assay Kits

Six laboratories participated in this study and validated seven Aβ40 assays (Table 1). Every commercial assay was validated by two or three experienced laboratories, which collaborated within the EU Joint Programme Neurodegenerative disease (JPND) BIOMARKAPD consortium. Additionally, the in-house Aβ40 assay was only tested by the developers. The following assays for quantification of Aβ40 concentrations were included: Aβ Peptide Panel I (4G8) kit [Cat#: K15199E, Meso Scale Discovery (MSD), Rockville, MD, USA^1^], amyloid-β (1-40) CSF ELISA [Cat#: RE59651, IBL-international (IBL), Hamburg, Germany^2^], ELISA Kit Human Aβ40 (Cat#: LNB0001, Invitrogen, Carlsbad, CA, USA^3^), Human Aβ40 Singleplex Bead Kit (Cat#: LHB3481, Novex, Invitrogen, Carlsbad, CA, USA, see text footnote 3), INNOTEST β-Amyloid (1-40) (Cat#: 81585, Fujirebio, Gent, Belgium^4^), β-amyloid (1-40) ELISA (Cat#: EQ 6511-9601-L, Euroimmun, Luebeck, Germany^5^), and an in-house Aβ40 ELISA [Ref. (18), VUmc, Amsterdam, The Netherlands]. Aβ40 assays of the same production batches from each vendor were directly distributed to the participating laboratories. The assays were performed manually according to the manufacturers’ protocols, some laboratories used plate washers for the washing steps. The laboratories tested all performance parameters according to the BIOMARKAPD SOPs (15, 16). Calculations of the analytical performance parameters were done using the corresponding Data Sheets S2 and S3 in Supplementary Material (15).

### Sensitivity

For the determination of the LLOQs, 16 blank samples were measured in one plate for each assay. The calibration curves were calculated using a four parameter logistic curve fit for all assays, which gave the optimal fit.

### Precision

Intra-assay variation (repeatability) was determined by analysis of samples (*n* = 15) in four replicates within one plate. Some deviations were made from the original protocol: in Lab #6, *n* = 3 samples instead of *n* = 15 samples were analyzed due to technical reasons. In Lab #2, *n* = 14 samples for the MSD assay and, in Lab #5, *n* = 16 samples for the Invitrogen assay were tested. The mean coefficient of variation (%CV) was calculated by averaging the CVs of all tested samples. A %CV <20% was defined as acceptable.

Inter-assay variation (intermediate precision) was measured to determine the variation of analyses between different days. To quantify inter-assay variation, samples with low, medium, and high concentrations were selected from the samples used for the intra-assay variation [quality control (QC) low, QC medium and QC high]. These samples were measured in duplicate in four different plates at identical positions in the assay plates on four different days. In Lab #2, three different plates were tested on three different days for the Invitrogen enzyme-linked immunosorbent assay (ELISA). The mean %CV was calculated for all samples. A %CV <20% was defined as acceptable.

Intra-plate variation was determined to explore the influence of different positions within a plate on the measured concentrations (stability of the plate). QC low, QC medium, and QC high samples were measured in four replicates at different positions of the plate (columns 3/4 vs. columns 11/12). The mean %CV was calculated for all samples. A %CV <20% was defined as acceptable.

### Dilutional Linearity

Three different CSF samples were used to perform the dilution linearity experiments. In Table S1 in Supplementary Material, the spiked Aβ40 calibrator concentration and the dilution factors used to serially dilute the samples per laboratory and assay are summarized. These samples were analyzed in duplicate. The dilutional linearity was calculated and expressed as follows:

$$\text{\%Linearity}=\frac{\left(\text{observed C * dilution factor}\right)}{\left(\text{previous observed C * previous dilution factor}\right)}\text{*100}$$

C=concentration(pg/mL)

A linearity between 80 and 120% was defined as acceptable.

To compare dilutional linearity for the kits from the various vendors and analyses by the different labs, we determined the length of dilution range in which dilutional linearity was within the acceptable range of 80–120%. This dilution range length was calculated using the following formula:

$$\text{Dilution range length}=\frac{\text{highest dilution factor in which the curve is linear}}{\text{lowest dilution factor in which the curve is still linear}}$$

Despite that protocols were distributed among the participating laboratories, some of them deviated from this protocol in the execution of the experiments to assess the dilutional linearity, since these laboratories did not spike the calibrator into the CSF samples. In addition, different interpretations of the distributed protocols may have resulted in the large differences in spiked Aβ40 calibrator concentrations by the various laboratories to assess dilutional linearity (Table S1 in Supplementary Material).

### Recovery

Five different CSF samples, measured in duplicate, were diluted according to manufacturers’ protocols and spiked with recombinant Aβ40 calibrator at three different levels. An overview of the spike low, medium, and high concentrations for each laboratory in every assay can be found in Table S2 in Supplementary Material. For neat samples, buffer without calibrator was added to the diluted CSF. Spike recoveries were calculated according to the formula:

$$\text{\% Recovery}=\frac{\left(\text{C spike sample-C neat sample}\right)}{\text{theoretical C spike}}\text{*100}$$

C=concentration(pg/mL)

A recovery between 80 and 120% was defined as acceptable.

### Parallelism

Five different CSF samples, with high endogenous protein concentrations, were serially diluted. Both reciprocal relative dilution factor and OD450 absorbance signals of the samples and calibrator were log-transformed to be able to use linear regression to calculate the slopes of the sample and calibrator curves. The slope of the linear parts of the log–log transformed calibrator and sample dilution series were compared to determine the degree of parallelism by calculating the “in range%” using the following formula:

$$\text{in range \%}=\frac{\text{slope of sample dilution series}}{\text{slope of calibration curve}}\text{*100}$$

A calculated in range% between 80 and 120% was defined as acceptable.

### Statistical Analysis

Bland–Altman plots were drawn to define if differences in results between assays were dependent on the concentration and to define the % deviation of each assay from the overall mean results for all clinical CSF samples. Correlation coefficients for comparison of results between vendors and between labs were performed by Spearman’s ρ. A *p*-value of 0.05 was considered significant.

## Results

### Sensitivity

An overview of the mean LLOQs per assay kit and for each laboratory per assay are presented in Table 2 and Table S3 in Supplementary Material, respectively. The mean LLOQs of the MSD and Fujirebio assays showed the largest variation between laboratories (CV = 99%), while the Euroimmun assay showed the smallest variation between laboratories (CV = 8%).

**Table 2:** Overview of analytical performance parameters.

		Sensitivity		Precision			Dilutional linearity range (pg/ml)	Parallelism (%P)	Intra-assay variation in clinical samples *n* = 20/laboratory (%CV)	Correlation coefficient (ρ) between laboratories
	
		LLOQ (pg/mL)		Intra-assay (%CV)	Inter-assay (%CV)	Intra-plate (%CV)
			
Vendor	Kit	Mean (SD)	%CV	Mean (SD)	Mean (SD)	Mean (SD)	Range (length)	Mean (SD)	Mean (SD)	Mean (SD)
MSD	Aβ peptide panel I (4G8)	65 (64)	99	7.1 (5.1)	13 (2.7)	6.1 (0.8)	7–2,825 (348)	92 (18)	5.8 (3.6)	0.86 (0.09)
IBL	Amyloid-β (1-40) CSF ELISA	41 (32)	79	3.0 (0.5)	10 (3.8)	3.2 (1.9)	15–480 (32)	100 (5.5)	2.4 (1.3)	0.93 (0.03)
Invitrogen	ELISA kit human Aβ40	28 (12)	40	7.3 (5.3)	18 (14)	7.0 (1.7)	2,500–97,656 (39)	189 (134)	3.7 (1.1)	0.91 (0.04)
Novex	Human Aβ40 singleplex bead kit	64 (36)	56	14 (1.9)	17 (8.0)	12 (5.4)	2,500–97,656 (39)	95 (12)	8.4 (1.1)	0.82 (–)
Fujirebio	INNOTEST β-amyloid (1-40)	1.8 (1.7)	99	5.1 (3.5)	13 (2.8)	4.0 (1.9)		73 (42)	3.6 (1.3)	0.80 (0.11)
Euroimmun	β-amyloid (1-40) ELISA	23 (1.9)	8	4.1 (2.5)	14 (6.0)	5.2 (3.6)	2,500–15,625 (6)	98 (6.7)	2.9 (1.8)	0.89 (0.03)
VUmc	In-house Aβ40	1,089 (−)	–	1.9 (−)	12 (−)	1.7 (−)	25– 125 (5)	93 (−)	1.1 (−)	–

*Aβ, amyloid-β; CSF, cerebrospinal fluid; ELISA, enzyme-linked immunosorbent assay; IBL, IBL-international; LLOQ, lower limit of quantification; MSD, Meso Scale Discovery; VUmc, VU university medical center; %CV, percentage coefficient of variation; %P, percentage parallelism*.

### Precision

The mean intra-assay CVs in all laboratories were below the predefined value of 20% for all assays (Table 2). However, it should be noted that the CVs of some individual samples were above this threshold for specific tests in single laboratories (Figure S1A in Supplementary Material).

The mean inter-assay CVs for all assays, but not for all individual laboratories, were below the predefined value of 20% (Table 2; Figure S1B in Supplementary Material).

The mean intra-plate CVs for all assays in all laboratories were below the predefined value of 20% (Table 2; Figure S1C in Supplementary Material). Only one individual sample in one laboratory showed an intra-plate CV above 20% (Figure S1C in Supplementary Material).

### Dilutional Linearity and Hook-Effect

No correct dilutional linearity data were obtained for the Fujirebio assay, Lab #2 and Lab #5. The remaining results are shown in Table 2 (overview) and Table S4 in Supplementary Material. The MSD assay showed an acceptable dilutional linearity between the predefined ranges of 80–120% over the longest dilution range, whereas this dilution range with acceptable linearity was the shortest for the Euroimmun and VUmc assays. Of note, the dilution range lengths with acceptable dilutional linearity may, in some cases, be longer than reported in Table S4 in Supplementary Material (as indicated by footnotes), since in these cases the highest or lowest dilution factor in which the curve was linear, corresponded to the highest or lowest dilution factor tested by the laboratory.

A hook effect, i.e., suppression of signal at concentrations above the upper limit of quantification, was observed for one sample tested in the Invitrogen ELISA by Lab #2 (between dilution factor 50 and 250; data not shown). Other samples tested by this and other laboratories neither showed a hook effect for the Invitrogen ELISA nor for any other assay (data not shown).

### Recovery

The results of the recovery (%R) experiments for all assays of different vendors are detailed in Table S5 in Supplementary Material. A large variation in recovery results between laboratories was observed for several assays. As a result, acceptable recovery within the predefined range of 80–120% for all three laboratories was only obtained for the IBL assay. In addition, acceptable recovery was obtained by individual laboratories for the MSD (1/3 laboratories), Invitrogen (1/3 laboratories), Novex (1/2 laboratories), Euroimmun (1/3 laboratories), and Fujirebio (1/3 laboratories) assays. Due to this large inter-laboratory variation, an overview of recovery results per vendor is not included in Table 2.

### Parallelism

Parallelism results are displayed in Table 2 (overview) and Table S6 in Supplementary Material. The mean percentage parallelism (%P) results for the assays of MSD, IBL, Novex, Euroimmun, and VUmc were within the predefined ranges of 80–120% (Table S6 in Supplementary Material), respectively: MSD (92%), IBL (100%), Novex (95%), Euroimmun (98%), and VUmc (93%). A large inter-laboratory variation for %P was observed using the Invitrogen and Fujirebio assays. For both these assays, the %P was within the predefined range for two out of three laboratories, while one laboratory per assay showed a strongly deviating result.

### Variation in Clinical Sample Concentrations between Laboratories

**Figure 1:**
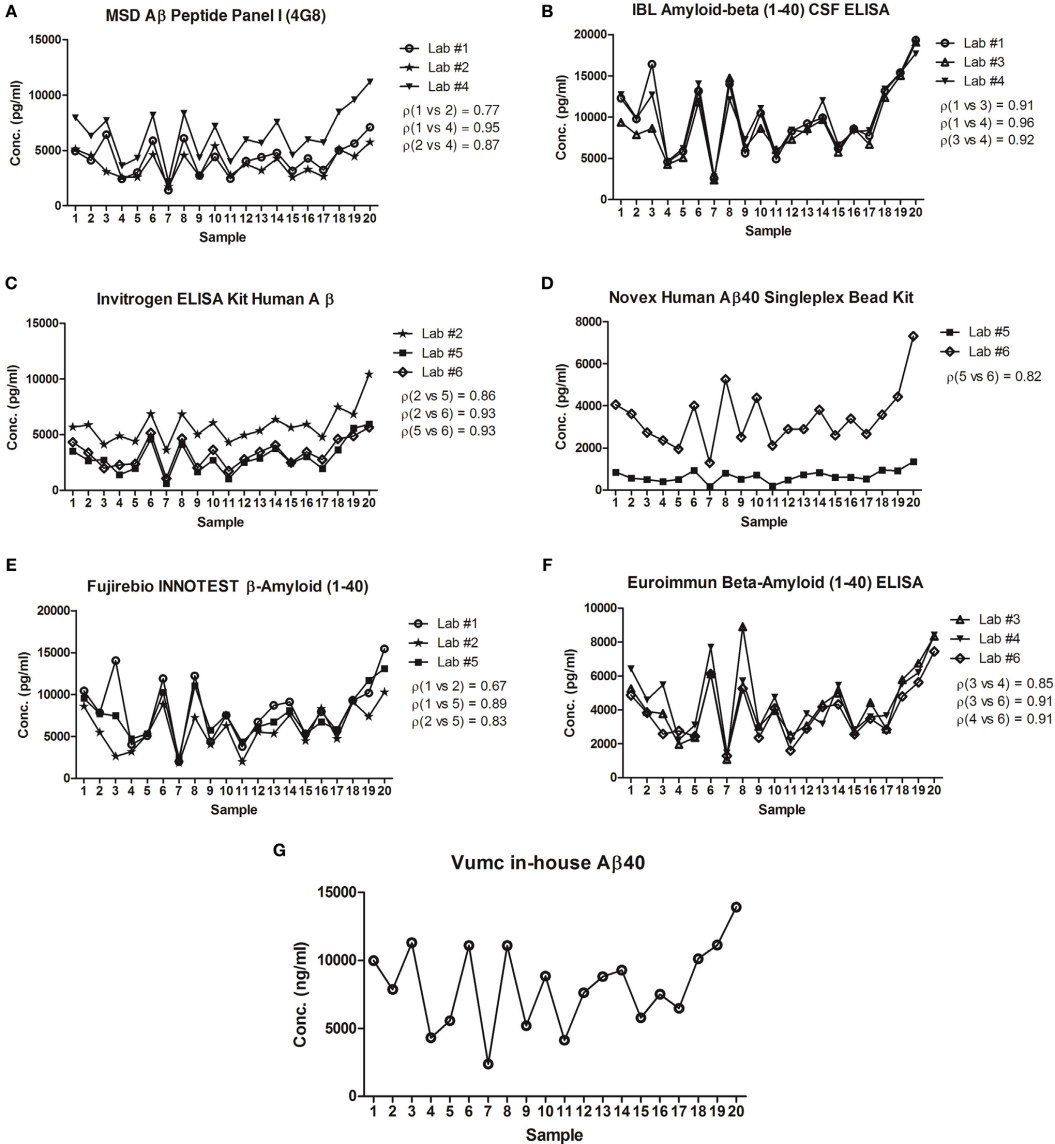
Correlation of concentrations in clinical cerebrospinal fluid (CSF) samples between laboratories. The mean concentrations of *n* = 20 samples tested in each laboratory for Meso Scale Discovery (MSD) Aβ peptide panel I (4G8) **(A)**, IBL amyloid-β (1-40) CSF ELISA **(B)**, Invitrogen ELISA kit human Aβ **(C)**, Novex Human Aβ40 Singleplex Bead Kit **(D)**, Fujirebio INNOTEST β-Amyloid (1-40) **(E)**, Euroimmun β-Amyloid (1-40) ELISA **(F)**, Vumc in-house Aβ40 **(G)** was plotted. The best correlation between laboratories was measured for concentrations using the IBL kit (mean ρ = 0.93). On the other hand, the lowest correlation was found in concentrations obtained by the Fujirebio assay (mean ρ = 0.80). ρ = Pearson’s correlation coefficient.

The intra-assay variation in Aβ40 concentrations was below 10% for all CSF samples (*n* = 20) in all laboratories for all different vendors, well below the predefined CV of 20% (Table 2). Per vendor, a strong correlation (ρ > 0.8) between sample Aβ40 concentrations assayed by the different laboratories was observed, except for the results by Lab #1 and Lab #2 that showed a lower correlation coefficient for the MSD (ρ = 0.77) and Fujirebio (ρ = 0.67) assays (Figure 1). The best mean correlation coefficient between laboratories was measured for Aβ40 concentrations using the IBL kit (ρ = 0.93) (Table 2). In terms of absolute concentrations, results for the Novex assay had the lowest similarity in mean values of the clinical samples between laboratories (Figure 1).

**Figure 2:**
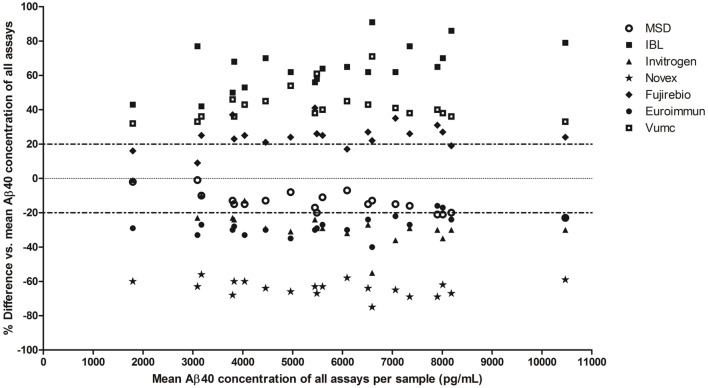
Variation in Aβ40 concentrations in clinical cerebrospinal fluid (CSF) samples between assays. Bland–Altman plot: the %difference (*y*-axis) in mean Aβ40 concentrations in each clinical CSF sample per assay (*n* = 20 samples, 7 assays) is compared to the mean Aβ40 concentration of all assays per sample. Each sample per assay is plotted on the *x*-axis at its mean Aβ40 concentration of all assays.

The mean Aβ40 concentrations of all CSF samples varied up from ~2,000 pg/mL for the Novex assay at the one extreme to ~9,500 pg/mL for the IBL assay at the other end. The %difference in Aβ40 concentrations per assay compared to the mean concentration of all assays per sample showed that the highest deviation was obtained by the IBL and Novex assays (mean deviation of 65 and −64%, respectively) (Bland–Altman plots in Figure 2). The lowest variation (within 20% deviation from the mean Aβ40 concentration of all assays per sample) was observed for the MSD assay.

## Discussion

The CSF Aβ42 levels reflect the extent of amyloid-β accumulation in the form of plaques in the brain. However, it has been shown in previous studies that the concentration of Aβ42 not only depends on the presence or absence of plaques but also may on the total turnover of Aβ peptides in the brain, resulting in different total Aβ concentrations in the CSF (11, 19–21). Besides, recent studies indicated that absorption of Aβ42 to collection and storage tubes can be overcome by calculation of the ratio of Aβ42/Aβ40, since both peptides will be absorbed to a similar degree (22, 23). The ratio of Aβ42/Aβ40 is helpful to correct levels of Aβ42 for the total amyloid production. In this study, we validated Aβ40 assays from six different vendors and one in-house developed assay for future use in clinical practice.

The assignment of the different assays to the laboratories was performed first based on the availability of the MSD and Luminex systems in the laboratories and second as random as possible, to overcome that two laboratories measured the same set of assays as a source of center bias. We have chosen to have the validation performed by up to three centers per assays, which we expect to provide sufficient insight into real-life performance of the assays and deemed to be cost-effective. We reasoned that testing all assays in a larger number of laboratories would not lead to dramatically different results for the assays than presented in this study.

The performance parameters of all assays were within the predefined ranges, except for the recovery results, for which no definite conclusions could be drawn due to large inter-laboratory variation, and the parallelism results of Invitrogen and Fujirebio assays, for each of which one out of three laboratories obtained results out of the predefined range. The variation between laboratories in the Aβ40 concentrations in the clinical samples was small in the duplicate measurements for each assay in every laboratory (%CV <10%). Furthermore, high mean correlation coefficients (ρ > 0.8) per vendor between sample concentrations were found for all assays. In terms of variation in absolute values between vendors, the least deviation was found for the MSD assay compared to the mean Aβ40 concentration of all assays per sample.

The LLOQs were slightly higher compared to the kit specifications provided by most vendors, but well below the ranges found in clinical samples. The IBL and Euroimmun assays are the exceptions, because the LLOQ was lower in our results compared to the kit specifications (data not shown). It should be noted, however, that the LLOQs are calculated differently by the vendors (mean + 2 to 3*SD), while we did use a more strict method of calculation (mean + 10*SD).

Although the mean precision data per assay were well below the predefined CV of 20% for all assays, if we would consider a more strict maximum of a mean CV of all laboratories below 15%, the results of Invitrogen and Novex assays are out of range.

The dilutional linearity description in the SOP gave room for many different interpretation options and should be revised. Some laboratories pre-diluted the CSF samples and, therefore, did not test the lower dilutions in which a hook effect still could be present. In the Invitrogen assay, a high variation was found, probably because Lab #6 tested lower steps of dilution (a factor 2.5) than the other two labs did (factor 4 and 5). Also, the amount of spiked antigen differed per laboratory; some laboratories did not spike antigen and, therefore, actually performed a parallelism experiment instead of the dilutional linearity experiment and had to be excluded from analysis for this parameter.

The recovery was out-of-range for the majority of the experiments. This could be due to the large variability between the laboratories in terms of preparation of the samples for the recovery experiments, e.g., the variation in concentrations chosen for spiking. In some laboratories, too high concentrations of Aβ40 were spiked, which resulted in an overflow or too low concentrations were spiked, resulting in very low precision of the result and thus low recovery. We have adapted the wording in the SOP to increase the compliance in execution (Supplemental Data for the improved version), e.g., by expanding on the choice of spikes and adding notes to standardize the spike concentrations in multicenter studies. Moreover, we added a proposal to include analysis of the actual spiked concentrations in reagent diluent for calculation instead of using a theoretical spiked value, in order to track possible dilution errors.

The parallelism results for the majority of the assays were within the predefined ranges, except the results of the Invitrogen and Fujirebio assays, because one laboratory was out of range. No explanation could be given for these two deviating results. However, with regard to this occurrence, to get more insight, it may be helpful to include more laboratories to test each validation parameter in future validation studies.

The IBL, Invitrogen, and Euroimmun Aβ40 assays seem to perform slightly better in terms of inter-laboratory comparisons, as shown by consistent high correlation coefficients for the clinical samples between laboratories. The mean Aβ40 concentrations of all CSF samples varied between the vendors. This is a hurdle in implementation of the ratio of Aβ42/Aβ40 in clinical practice to improve diagnostic accuracy (9–13). As long as this variation between assays is not overcome by the use of certified reference methods and materials, which are being developed (24), cut-offs will depend on the combination of assays used at every site. Usage of automated platforms instead of manual performance of biomarker measurements could mitigate inter-laboratory variations.

In conclusion, all Aβ40 assays perform generally well and are promising for implementing in clinical practice. We showed some deviating results in specific parameters, but these are more likely the result of inter-laboratory variation and the lack of reference materials. Furthermore, of note is that the absolute Aβ40 concentrations in clinical CSF varied between assays. Therefore, the assays could be improved by the availability of certified reference materials to calibrate kits as well as usage of automated platforms. The upcoming availability of Aβ40 assays in a European Conformity (CE)-certified format is an important first step for their routine implementation in Europe.

## Ethics Statement

Participants or their legal representatives gave informed consent. The study conforms with The Code of Ethics of the World Medical Association (Declaration of Helsinki) (World_Medical_Association 1964). The biobank containing the coded patient samples was approved by the Local Ethical committee of the VU University Medical Center Amsterdam.

## Author Contributions

LWD: performed data analysis and drafted the manuscript. LK: supervision of data collection and study design, critical review of the manuscript. MK-S: study design and coordination, data collection, and analysis. HK, SE, AP-L, SL, MV, and EV: supervision of data collection and study design, critical review of the manuscript. AV: data collection, critical review of the manuscript. HS, HT, and AF: data collection and analysis, critical review of the manuscript. EV: supervision of data collection and study design, critical review of the manuscript. CT: study design and coordination, data analysis, critical review of the manuscript.

## Conflict of Interest Statement

SE was/is consultant for and/or received research funding from Janssen Pharmaceutica, ADx NeuroSciences, Innogenetics/Fujirebio Europe, Lundbeck, Pfizer, Novartis, UCB, Roche diagnostics, Nutricia/Danone, and Eli Lilly. MV serves on an advisory board for Roche Diagnostics. CT served on the advisory board of Fujirebio and Roche, received research consumables from Euroimmun, IBL, Fujirebio, Invitrogen, and Meso Scale Discovery, and performed contract research for IBL, Shire, Boehringer, Roche, and Probiodrug. All other authors declare no conflict of interest.

## Footnotes

^1^www.mesoscale.com.

^2^www.ibl-international.com.

^3^www.thermofisher.com.

^4^www.fujirebio-europe.com.

^5^www.euroimmun.com.

## Abbreviations

Aβ, amyloid-β peptide; CSF, cerebrospinal fluid; SOP, standard operating procedure; AD, Alzheimer’s disease; t-tau, total tau protein; p-tau, tau phosphorylated at threonine 181; MCI, mild cognitive impairment; PET, positron emission tomography; LLOQs, lower limits of quantification; 4PL, four parameter logistic; %CV, coefficient of variation; QC, quality control; ELISA, enzyme-linked immunosorbent assay; CE, European Conformity.
