# Supplementary material for: Multicenter Analytical Validation of Aβ40 Immunoassays
Source: Front Neurol. 2017 Jul 3;8:310. doi: 10.3389/fneur.2017.00310 (PMC5497061; doi:10.3389/fneur.2017.00310)
Supplement: Supplementary file 1 [file Supplemental_Data.PDF]

Multicenter analytical validation of Aβ40 immunoassays

Linda J.C. van Waalwijk van Doorn[1,2*], Luka Kulic[3], Marleen J.A. Koel-Simmelink[4], H. Bea Kuiperij[1,2], Alexandra A.M. Versleijen[2], Hanne Struyfs[5], Harry A.M. Twaalfhoven[4], Anthony Fourier[6], Sebastiaan Engelborghs[5,7], Armand Perret-liaudet[6], Sylvain Lehmann[8], Marcel M. Verbeek[1,2], Eugeen Vanmechelen[9], Charlotte E. Teunissen[4]

[1] Department of Neurology, [2] Department of Laboratory Medicine, Radboud University Medical Center, Radboud Alzheimer Centre, Donders Institute for Brain, Cognition and Behaviour, Nijmegen, the Netherlands

[3] Institute for Regenerative Medicine (IREM), University of Zurich, Schlieren, Switzerland

[4] Neurochemistry Laboratory and Biobank, Department of Clinical Chemistry, VU University Medical Center, Neurocampus Amsterdam, the Netherlands

[5] Reference Center for Biological Markers of Dementia (BIODEM), Institute Born-Bunge, University of Antwerp, Antwerp, Belgium

[6] Neurobiology Laboratory, Centre for Memory Resources and Research (CMRR), Groupement Hospitalier Est (GHE), Hôpitaux de Lyon, Université Lyon 1, CNRS UMR5292, INSERM U1028, Lyon, France

[7] Memory Clinic and Department of Neurology, Hospital Network Antwerp (ZNA) Middelheim and Hoge Beuken, Antwerp, Belgium

[8] CHU de Montpellier and Université de Montpellier, IRMB, Laboratoire de Biochimie Protéomique Clinique, Montpellier, France

[9] R&D, ADx NeuroSciences, Ghent, Belgium

Supplemental Data

Improvements for SOP

Dilution Linearity

Dilution linearity is performed to demonstrate that a sample with a concentration above the ULOQ can be diluted to a concentration within the working range and still give a reliable result. In other words, it determines to which extent the dose-response of the analyte is linear in a particular diluent within the range of the standard curve. Thereby, dilution of samples should not affect the accuracy and precision. At the same time, the presence of a hook effect, i.e., suppression of signal at concentrations above the ULOQ, is investigated.

A few helpful notes:

- Make sure you make your dilution series covering the whole range, starting above the highest standard (or at least below ULOQ) until below blank (or at least below LLOQ). Do not pre-dilute your samples, otherwise you miss the lower dilutions in which a possible hook effect could be present.
- In a multicenter study it is important to agree on forehand on e.g. the dilution factor of the assay to be evaluated, and the concentrations of recombinant protein that will be spiked to the samples, in order to compare the same assay in different laboratories. If you do not spike recombinant protein to your samples, you actually perform a parallelism experiment instead of the dilutional linearity experiment.

Procedure

1. Spike three **undiluted** samples with calibrator stock solution, as high as possible. Note: if possible, spike **undiluted** samples with 100- to 1000-fold the concentration at ULOQ using the calibrator stock solution. Spiking with a high concentration (at least

to an end-concentration above the highest standard) of recombinant antigen is needed to observe whether a hook effect is present or not. Biological samples can also be diluted less than the prescribed concentration, if an assay allows to.

2. Make serial dilutions of the spiked samples, using sample diluent in small vials until the theoretical concentration is below LLOQ. Check that the expected concentrations are beyond the ULOQ and LLOQ of the standard curve, to be able to define the maximum dilution range. Note: the dilution should be performed using vials and not directly in the wells of the ELISA plate.

3. Analyze the serial dilutions in duplicates and compensate for the dilution factor.

4. Calculate for each sample the mean concentration for the dilutions that fall into the range of LLOQ and ULOQ. Moreover, calculate for each sample the %Recovery for the calculated concentration at each dilution. Note: the calculated concentration for a dilution that fall into the range of LLOQ and ULOQ should be within the acceptance criteria for the precision defined in the "SOP for fit-for-purpose" as should the calculated SD. Also, plot the signal against the dilution factor to investigate if the signal is suppressed at much higher concentrations than the ULOQ of the sample ("hook effect").

Dilution linearity should not be confused with linearity of quantitative measurement procedures as defined by CLSI, which concerns the linearity of the calibration curve.

Recovery

The recovery of an analyte in an assay is the detector response obtained from an amount of the analyte added to and extracted from the biological matrix, compared to the detector response obtained for the true concentration of the analyte in solvent. A spike recovery test is conducted to investigate if the concentration-response relationship is similar in the calibration curve and the samples. A bad outcome of the test suggests that there are differences between the sample matrix and calibrator diluent that affects the response in signal. Data obtained from this study could help to find a diluent mimicking the biological sample in which the calibrator and the native protein give the comparable detector signals all along the measuring range.

<u>A helpful note:</u>

- In a multicenter study it is important to agree on beforehand on the concentrations of recombinant protein that will be spiked to the samples for a certain assay in order to compare the same assay in different laboratories.

Procedure

1. Collect five samples with known concentration and divide each sample into 4 aliquots.

2. Spike three of the aliquots, using calibrator stock solution, to expected concentrations that are evenly distributed over the linear range of the standard curve (low, medium, high). Note: all additions should be in the same volume, preferable <10% of the sample volume. The same volume of sample-free calibrator diluent must also be added to the neat sample (fourth aliquot) to compensate for the dilution.

   Important: different spiking concentrations should be used to investigate possible dependency on the amount of added substance. The theoretical concentration in the

spiked samples should be lower than the ULOQ. The low spike should be slightly higher than the lowest reliable detectable concentration. It should be stressed that when spiking too high concentrations of an analyte, it will result in an overflow. If too low concentrations are spiked, it will result in very low precision of recoveries. Note: alternatively, samples can be spiked after dilution if there is limited availability of the calibrator and high working dilutions.

3. Spike the three tested concentrations (low, medium, high) also to reagent diluent (instead of using theoretical spike concentrations), to correct for analytical variation. Additionally, this is supported by the fact that subtraction methods are preferred over addition methods (Marcelletti *et al.* 2015).

4. Analyze both the neat and spiked samples in the same run. Dilute each sample as advised for each assay to be used.

5. Calculate the recovery using the formula below. Note: acceptance range for the recovery is usually 80-120%.

   Spike recoveries were calculated according to the formula:

$$\%\, Recovery = \frac{(C\ spiked\ sample - C\ neat\ sample)}{C\ spike\ in\ diluent} * 100$$

C = concentration (pg/mL)

References

Marcelletti, J. F., Evans, C. L., Saxena, M. and Lopez, A. E. (2015) Calculations for Adjusting Endogenous Biomarker Levels During Analytical Recovery Assessments for Ligand-Binding Assay Bioanalytical Method Validation. *The AAPS journal,* **17,** 939-947.

Supplemental Table 1: Dilutional linearity, preparation of samples (n=3 / laboratory)

Vendor	Preparation	Lab #1	Lab #2	Lab #3	Lab #4	Lab #5	Lab #6
**MSD**	Antigen added[a]	29,100	0	-	34,920	-	-
	Dilution factor	7	6	-	7	-	-
**IBL**	Antigen added[a]	0	-	3,000	0	-	-
	Dilution factor	3	-	2	4	-	-
**Invitrogen**	Antigen added[a]	-	0	-	-	0	1,000,000
	Dilution factor	-	5	-	-	4	2.5
**Novex**	Antigen added[a]	-	-	-	-	0	1,000,000
	Dilution factor	-	-	-	-	2	2.5
**Fujirebio**	Antigen added[a]	0	0	-	-	0	-
	Dilution factor	5	4	-	-	4	-
**Euroimmun**	Antigen added[a]	-	-	6,000	0	-	1,000,000
	Dilution factor	-	-	2	4	-	2.5
**VUmc**	Antigen added[a]	250,000	-	-	-	-	-
	Dilution factor	5	-	-	-	-	-

[a] *in undiluted CSF, in pg/mL.*

Supplemental Table 2: Recovery, preparation of samples (n=5 / spike / laboratory)

Vendor	Spike[a]	Lab #1	Lab #2	Lab #3	Lab #4	Lab #5	Lab #6
**MSD**	Low	200	1,500	-	400	-	-
	Medium	1,600	3,000	-	3,200	-	-
	High	8,000	4,500	-	16,000	-	-
**IBL**	Low	50	-	100	1,000	-	-
	Medium	200	-	400	4,000	-	-
	High	800	-	1,000	16,000	-	-
**Invitrogen**	Low	-	125[b]	-	-	10	175
	Medium	-	250[b]	-	-	50	289
	High	-	375[b]	-	-	200	400
**Novex**	Low	-	-	-	-	106	1,750
	Medium	-	-	-	-	850	2,890
	High	-	-	-	-	3,400	4,000
**Fujirebio**	Low	9	20[c]	-	-	17	-
	Medium	47	39[c]	-	-	95	-
	High	189	59[c]	-	-	237	-
**Euroimmun**	Low	-	-	200	1,050	-	175
	Medium	-	-	400	4,200	-	289
	High	-	-	600	16,800	-	400
**VUmc**	Low	1,000	-	-	-	-	-
	Medium	3,000	-	-	-	-	-
	High	10,000	-	-	-	-	-

[a] *Spike low, medium and high are in pg/mL.*
[b] *Two samples had a spike low, medium and high of half of the indicated values.*
[c] *Two samples had different spiked concentrations (low: 50, medium: 100, high: 150).*

Supplemental Table 3: Sensitivity (LLOQ in pg/mL)

Vendor	Range	Lab #1	Lab #2	Lab #3	Lab #4	Lab #5	Lab #6	Mean	SD	%CV
**MSD**	4 – 15,000	44	137	-	14	-	-	65	64	99
**IBL**	0 – 1,900	74	-	40	9.1	-	-	41	32	79
**Invitrogen**	1 – 500	-	42	-	-	20	24	28	12	40
**Novex**	0 – 5,000	-	-	-	-	89	39	64	36	56
**Fujirebio**	5 – 1,000	3.5	0.1	-	-	1.2	-	1.8	1.7	99
**Euroimmun**	0 – 800	-	-	21	25	-	24	23	1.9	8
**VUmc**	0 – 25,000	1,089	-	-	-	-	-	1,089	-	-

Key: LLOQ, lower limit of quantification; SD, standard deviation, %CV, coefficient of variation.

Supplemental Table 4 : Dilutional linearity

	Lab #1				Lab #3				Lab #4				Lab #6			
**Vendor**	**Spiked antigen (pg/mL)**	**Dilution range**	**Dilution range length**	**Mean %L**	**Spiked antigen (pg/mL)**	**Dilution range**	**Dilution range length**	**Mean %L**	**Spiked antigen (pg/mL)**	**Dilution range**	**Dilution range length**	**Mean %L**	**Spiked antigen (pg/mL)**	**Dilution range**	**Dilution range length**	**Mean %L**
**MSD**	29,100	7 – 2,401	343	105%	-	-	-	-	34,920	8 – 2,825	353	100%	-	-	-	-
**IBL**	NA	NA	NA	NA	3,000	15 – 480	32[a]	94%	NA	NA	NA	NA	-	-	-	-
**Invitrogen**	-	-	-	-	-	-	-	-	-	-	-	-	1,000,000	2,500 – 97,656	39[b]	98%
**Novex**	-	-	-	-	-	-	-	-	-	-	-	-	1,000,000	2,500 – 97,656	39[b]	97%
**Euroimmun**	-	-	-	-	6,000	60 – 480	8[b]	98%	NA	NA	NA	NA	1,000,000	2,500 – 15,625	6	92%
**VUmc**	250,000	25 – 125	5	96%	-	-	-	-	-	-	-	-	-	-	-	-

Key: %L, percentage linearity. The spiked antigen in undiluted CSF is indicated. The dilutional linearity was expressed as the dilution range (pg/mL) and the length of the dilution range in which the mean %L calculated from three samples was within the pre-defined range of 80-120%. NA, not applicable: no antigen spiked, nota bene the Fujirebio assay, Lab #2 and Lab #5 were left out of the table, because no antigen was spiked in any experiment.

[a] *Lowest dilution factor in which the curve is linear corresponds to the lowest dilution tested.*

[b] *Highest dilution factor in which the curve is linear corresponds to the highest dilution tested.*

Supplemental Table 5: Recovery

Vendor	Lab #1	Lab #2	Lab #3	Lab #4	Lab #5	Lab #6	Mean	SD
**MSD**	85%	66%	-	76%	-	-	76%	10%
**IBL**	102%	-	97%	98%	-	-	99%	3%
**Invitrogen**	-	Technical failure	-	-	6%	88%	47%	58%
**Novex**	-	-	-	-	6%	88%	47%	58%
**Fujirebio**	95%	71%	-	-	11%	-	59%	43%
**Euroimmun**	-	-	123%	13%	-	110%	82%	60%
**VUmc**	71%	-	-	-	-	-	71%	-

Key: SD, standard deviation. Recovery values are expressed as mean % per laboratory..

Supplemental Table 6: Parallelism

Vendor	Lab #1	Lab #2	Lab #3	Lab #4	Lab #5	Lab #6	Mean	SD
**MSD**	110%	74%	-	91%	-	-	92%	18%
**IBL**	100%	-	94%	105%	-	-	100%	6%
**Invitrogen**	-	344%	-	-	118%	105%	189%	134%
**Novex**	-	-	-	-	86%	103%	95%	12%
**Fujirebio**	91%	104%	-	-	25%	-	73%	42%
**Euroimmun**	-	-	90%	101%	-	102%	98%	7%
**VUmc**	93%	-	-	-	-	-	93%	-

Key: SD, standard deviation. Parallelism values are expressed as mean % per laboratory.

Supplemental Table 7: Distribution of samples per assay versus the mean (reference)

Sample	Mean (reference)	MSD	IBL	Invitrogen	Novex	Fujirebio	Euroimmun	Vumc
**1**	7061	-15	62	-36	-65	35	-22	41
**2**	5599	-11	64	-29	-63	25	-27	40
**3**	6594	-13	91	-55	-75	22	-40	71
**4**	3170	-10	42	-10	-56	25	-27	36
**5**	3796	-13	50	-23	-68	37	-30	46
**6**	7902	-21	65	-30	-69	31	-16	40
**7**	1795	-2	43	-1	-60	16	-29	32
**8**	8011	-21	70	-35	-62	27	-17	38
**9**	3827	-15	68	-24	-60	23	-28	36
**10**	6091	-7	65	-32	-58	17	-30	45
**11**	3090	-1	77	-23	-63	9	-33	33
**12**	4960	-8	62	-31	-66	24	-35	54
**13**	5486	-20	58	-29	-67	26	-29	61
**14**	6511	-15	62	-27	-64	27	-24	43
**15**	4038	-15	53	-13	-60	25	-33	43
**16**	5447	-17	56	-24	-63	41	-30	38
**17**	4458	-13	70	-29	-64	21	-30	45
**18**	7346	-16	77	-29	-69	26	-27	38
**19**	8183	-20	86	-30	-67	19	-24	36
**20**	10468	-23	79	-30	-59	24	-23	33

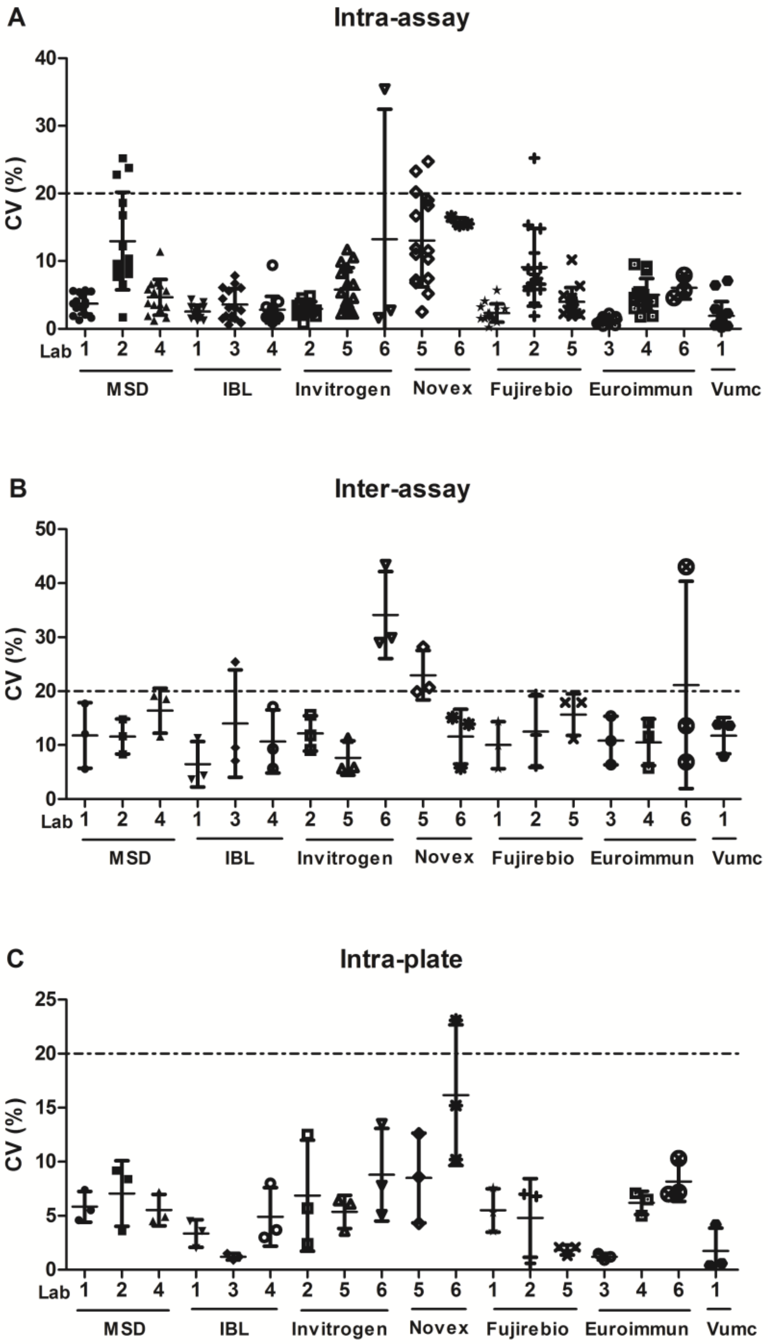

Supplemental Figure 1: Precision of Aβ40 assays defined as intra-assay, inter-assay and intra-plate variation

The intra-assay CV **(A)**, inter-assay CV **(B)** and intra-plate CV **(C)** for Aβ40 concentrations of assays from different vendors between the laboratories (numbers on x-axis). The mean ± standard deviation (SD) is indicated per laboratory. CV (%) = Coefficient of variation, acceptable pre-defined value of < 20%.
